# Supplementary material for: ATP sensing in living plant cells reveals tissue gradients and stress dynamics of energy physiology
Source: eLife. 2017 Jul 18;6:e26770. doi: 10.7554/eLife.26770 (PMC5515573; doi:10.7554/eLife.26770)
Supplement: Supplementary file 1. — DOI: http://dx.doi.org/10.7554/eLife.26770.023 [file elife-26770-supp1.docx]

**Supplementary file 1: Oligonucleotides for generation of expression constructs**

| **Name** | **Sequence** | **Purpose** |
| --- | --- | --- |
| 2691 | GGGGACAAGTTTGTACAAAAAAGCAGGCTT | PCR-amplify Gateway-compatible ATeam1.03-nD/nA from pENTR1A:ATeam1.03-nD/nA |
| 2692 | GGGGACCACTTTGTACAAGAAAGCTGGG |  |
| 3036 | GGGGACAAGTTTGTACAAAAAAGCAGGCTCCACC**ATGGCTTCTCGGAGGCTTCT** | PCR-amplify the *Nicotiana plumbaginifolia* ß-ATPase target peptide (**BOLD**) preceeded by a Gateway attB1 site (UNDERLINED) and followed by an overlap for fusion with the 5’ end of ATeam1.03-nD/nA (*ITALICS*) |
| 3042 | *CTCCTCGCCCTTGCTCAC***ACCAGCGCCGGTGAACTC** |  |
| 3043 | **GAGTTCACCGGCGCTGGT***GTGAGCAAGGGCGAGGAG* | PCR-amplify the 5’ end of ATeam1.03-nD/nA (*ITALICS*) preceeded by an overlap for fusion with the *Nicotiana plumbaginifolia* ß-ATPase target peptide (**BOLD**) |
| 3044 | *CCATCTCGATATCAGCGTCG* |  |
| 3045 | *CGACGCTGATATCGAGATGG* | PCR-amplify the 3’ end of ATeam1.03-nD/nA (*ITALICS*) followed by a Gateway attB2 site (UNDERLINED) |
| 3046 | GGGGACCACTTTGTACAAGAAAGCTGGGTG*TTACTCGATGTTGTGGCGGAT* |  |
| 3047 | GGGGACAAGTTTGTACAAAAAAGCAGGCTCC | Primers comprising the Gateway attB sites to amplify the fusion of *Nicotiana plumbaginifolia* ß-ATPase target peptide and ATeam1.03-nD/nA |
| 3048 | GGGGACCACTTTGTACAAGAAAGCTGGGTG |  |
| ATeam-BamHI-F | ATTAGGATCCTCGAGTATGGTGAGCAAGGGC | PCR-amplify ATeam1.03-nD/nA for subcloning into pENTR/D-TOPO; restriction sites are underlined |
| ATeam-XbaI-R | TCCGTCTAGATTACTCGATGTTGTGGCGGATCT |  |
| TKTPsp-NdeI-F | CATTCATATGATGGCGTCTTCTTCTTCTCT | PCR-amplify *Nicotiana tabacum* transketolase target peptide for subcloning into pENTR/D-TOPO; restriction sites are underlined |
| TKTPsp-PstI-R | ATTACTGCAGCGCAGTCTCAGTTTTCTCTAT |  |
